# Overexpression of B7-H3 Is Associated With Poor Prognosis in Laryngeal Cancer

**DOI:** 10.3389/fonc.2021.759528

**Published:** 2021-12-06

**Authors:** Yixuan Li, Qian Cai, Ximing Shen, Xiaoting Chen, Zhong Guan

**Affiliations:** ^1^ Department of Otolaryngology, Sun Yat-sen Memorial Hospital, Sun Yat-sen University, Guangzhou, China; ^2^ Department of Pathology, Sun Yat-sen Memorial Hospital, Sun Yat-sen University, Guangzhou, China

**Keywords:** B7-H3, biomarker, LSCC - laryngeal squamous cell carcinoma, diagnosis, prognosis

## Abstract

The immune checkpoint molecule, B7-H3, which belongs to the B7 family, has been shown to be overexpressed in various cancers. Its role in tumors is not well defined, and many studies suggest that it is associated with poor clinical outcomes. The effect of B7-H3 on laryngeal cancer has not been reported. This study investigated the expression of B7-H3 in laryngeal squamous cell carcinoma (LSCC), and its relationship with clinicopathological factors and prognosis of LSCC patients. The gene expression quantification data and clinical data of LSCC retrieved from The Cancer Genome Atlas (TCGA) and Gene Expression Omnibus (GEO) database were analyzed to determine the diagnostic and prognostic roles of B7-H3. Quantitative real-time polymerase chain reaction (qRT-PCR) was then performed to determine the gene expression level of B7-H3 between LSCC tissues and paired normal adjacent tissues. In addition, TCGA RNA-seq data was analyzed to evaluate the expression level of B7 family genes. Next, the protein expression of B7-H3 and CD8 in LSCC was determined using immunohistochemistry and immunofluorescence. qRT-PCR results showed that the expression level of B7-H3 mRNA was significantly higher in LSCC tissues than in adjacent normal tissues. Similar results were obtained from the TCGA analysis. The expression of B7-H3 was significantly associated with T stage, lymph node metastasis, and pathological tumor node metastasis (TNM) stage, and it was also an independent factor influencing the overall survival time (OS) of patients with LSCC. In addition, B7-H3 was negatively correlated with CD8^+^T cells. These results show that B7-H3 is upregulated in LSCC. Therefore, B7-H3 may serve as a biomarker of poor prognosis and a promising therapeutic target in LSCC.

## Introduction

Laryngeal cancer is ranked as the second most prevalent head and neck tumor after lip and oral cavity cancers worldwide (1). A previous study reported that the incidence and number of deaths due to laryngeal cancer increased steadily from 1997 to 2017 worldwide (2). The main causes of deaths due to this cancer are tumor recurrence and distant metastasis. To date, there is no effective treatment method for advanced laryngeal cancer. This calls for in-depth research to identify new diagnostic biomarkers and therapeutic targets in laryngeal cancer.

As a member of the newly discovered immunomodulatory protein B7 family, B7-H3 has been found to play an important role in a variety of tumors and immune diseases. One study reported that B7-H3 can regulate activation of T cells and promote secretion of inflammatory factors (3). Recent studies have also confirmed that B7-H3 has an immunosuppressive function in several cancers (4, 5), including non-small cell lung cancer (6), breast cancer (7), and other tumors. In these cancers, it affects the prognosis of patients. Moreover, evidence suggests that overexpression of B7-H3 is closely associated with tumor cell apoptosis, cell metabolism, tumor angiogenesis, tumor drug resistance, and other aspects (8–11). However, the role of B7-H3 in laryngeal cancer has not been elucidated.

Therefore, this study investigated the expression level of B7-H3 in laryngeal squamous cell carcinoma (LSCC) tissues, and explored its association with clinicopathological features, cancer prognosis, and tumor-infiltrating CD8^+^ T cells. In addition, clinical results were combined with data retrieved from The Cancer Genome Atlas (TCGA) to determine the functions of B7-H3 on LSCC. To the best of our knowledge, this is the first study that has elaborated the role of B7-H3 in laryngeal cancer.

## Materials and Methods

### Data Retrieval and Determination of Differential Expression of B7 Family mRNAs

Clinical characteristics and gene expression data of LSCC patients of the HTSeq-FPKM type were acquired from *TCGA* (http://tcga-data.nci.nih.gov/tcga/) and *GEO* (https://www.ncbi.nlm.nih.gov/geo/) on 4^th^ May 2021. The mRNA expression data of LSCC patients was also downloaded from the TCGA database and used to determine differentially expressed genes (DEGs) of B7 family in LSCC tissues. All samples were analyzed using the bioconductor package of edgeR. Results were visualized using gplots packages in R software (version 3.0.1.2). The DEGs were identified using |log2-fold change| ≥ 1.0 as the cut-off. Next, the survival R package was used to generate Kaplan-Meier survival curves of the DEGs, with *P*< 0.05 being the cut-off for statistical significance.

### Patients and Clinical Samples

A total of 122 laryngeal cancer patients who underwent surgery between December 2012 and November 2015 were included in this study. The included patients met the following inclusion criteria: underwent surgery as the first treatment, preoperative pathological diagnosis was laryngeal squamous cell carcinoma, and did not receive preoperative radiotherapy, chemotherapy or other treatments. Exclusion criteria were: patients with other tumors or recurrent tumors and patients with other severe diseases or autoimmune disease. All patients were followed up for at least five years. During surgery, paired fresh laryngeal cancer specimens and normal adjacent tissues were collected from 12 patients. Patient clinicopathological variables were collected by reviewing medical records. Data extracted included: age, sex, primary tumor site, tumor differentiation, TNM stage, smoking index, and alcohol consumption. The TNM stage was defined according to the American Joint Committee on Cancer (*AJCC*). Overall survival (OS) was defined as the period from the date of surgery to the time of death or last follow-up, which was 1^st^ November 2020. This study was approved by the institutional ethical review board of Sun Yat-Sen Memorial Hospital and signed informed consent form was obtained from all patients before the study.

### RNA Extraction and Real-Time Quantitative PCR

Total RNA was extracted from homogenized frozen tissues using TRIzol reagent (Guangzhou IGE Bio.) and reverse transcribed to cDNA using a RevertAid First Strand cDNA Synthesis Kit (Guangzhou IGE Bio.) according to the manufacturer’s instructions. Next, quantitative real-time polymerase chain reaction (qRT-PCR) was performed on a Roche LightCycler 480 Real-Time PCR system using a SYBR Premix Ex Taq (Takara) according to the manufacturer’s instructions. The reaction protocol was as follows:1 min at 95°C, 40 cycles of 10 seconds at 95°C and 12 min at 60°C, and finally a 65°C to 95°C ramp up to determine the melting curve. Relative mRNA level of B7-H3 was normalized to that of GAPDH mRNA. The relative mRNA expression was calculated using the comparative 2^-△△Ct^ method.

### Immunohistochemistry

A total of 152 wax samples, including 122 LSCC samples and 30 adjacent normal tissues, were obtained from the pathology department of Sun Yat-sen Memorial Hospital. All samples were deparaffinized and rehydrated through a series of alcohol baths: 100% xylene (2×20 min), 100% ethanol (2×5 min), 95% ethanol, 80% ethanol, 75% ethanol and H_2_O (1×2 min each). Next, the samples were incubated in 10 mM sodium citrate buffer (pH 6.0) for antigen retrieval, followed by incubation with goat anti-human B7-H3 antibody (Cell Signaling Technology, 1:50) overnight at 4°C. Finally, 24 samples were further incubated with CD8 antibody (Cell Signaling Technology, 1:100) overnight at 4°C.

### Evaluation of Immunohistological Staining

The staining intensity of B7-H3 was evaluated by two experienced pathologists using an independent double-blind method. Ten high-power fields (magnification×200) were selected to examine the positively stained area. The intensity of stained area was scored as follows: negative (0), weak (1), moderate (2), and strong (3). In addition, the percentage of positively stained cells was scored as: 0% to 5% (0), 6% to 25% (1), 26% to 50% (2), and >50% (3). To calculate the weighted scores for each section, the percentage of stained cells was multiplied with the staining intensity scores. Subsequently, a score of 4 was used as the cut-off to distinguish low B7-H3 expression low (<4) from high expression (≥4). The number of CD8^+^ T-cells in LSCC tissues was counted in three randomly selected tumor areas at a magnification of ×200. The average count was finally.

### Double Immunofluorescence Staining

Paraffin‐embedded (FFPE) slides of LSCC tissues were dewaxed, rehydrated, and incubated in the retrieval buffer solution for antigen retrieval as described above. Next, the samples were incubated with a mixture of two primary antibodies [B7-H3 (goat antibody) and CD8 (rabbit antibody)] overnight at 4°C, followed by a mixture of FITC- and Cy3-conjugated secondary antibodies. The cell nuclei were counterstained blue with a DAPI solution. Images were acquired from at least three randomly high‐power fields using the Olympus BX51 microscope at the appropriate excitation wavelength for the fluorophore.

### Statistical Analysis

All statistical analyses were performed using SPSS version 25.0 (IBM, Armonk New York). Data are presented as the mean ± S.E.M. Wilcoxon rank sum test was used to compare mRNA expression level between LSCC and noncancerous tissues. Categorical variables were analyzed using Fisher’s exact or Pearson’s chi-squared test. Moreover, the overall survival (OS) curve of patients was estimated using the Kaplan-Meier method. Univariate and multivariate analysis for hazard estimation was done using Cox regression. A *P* < 0.05 was considered statistically significant, and GraphPad Prism software version 8.0 was used to generate the figures.

## Results

### Identification of Differentially Expressed Genes of B7 Family in LSCC Tissues

**Figure 1:**
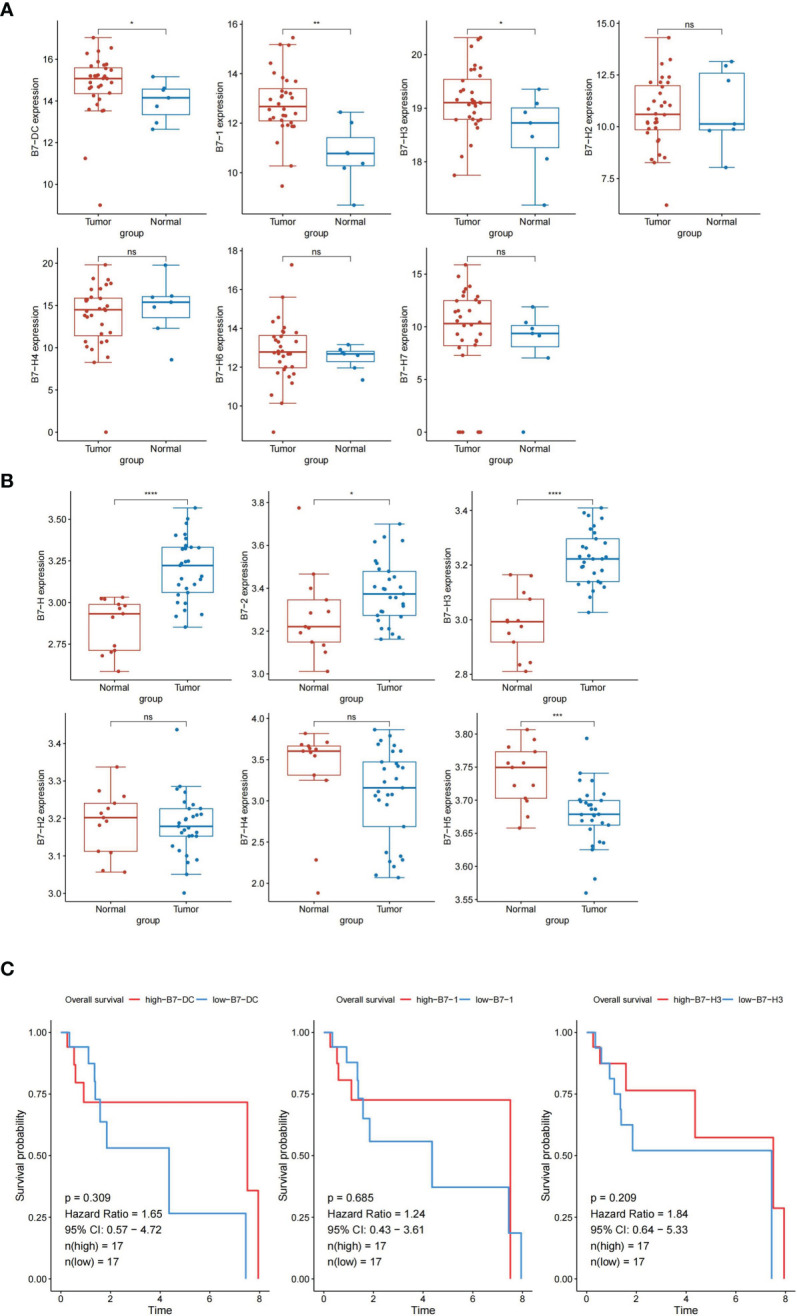
The expression levels of B7 family members in LSCC samples and normal tissues. The data was obtained from TCGA database **(A)** and GEO database **(B)**. ns, not significant, *p < 0.05, **p < 0.01, ***p < 0.001,****p < 0.0001. Overall survival of LSCC patients grouped based on *B7-1*, *B7-DC*, and *B7-H3* expression level by Kaplan-Meier analysis **(C)**. *p* < 0.05 considered as significant.

To identify the association of B7 family genes with prognosis of LSCC, the RNA-Seq data of 32 LSCC samples and 7 normal tissues were obtained from the TCGA database. The mRNA expression profiles of seven B7 family genes were also extracted from the database and are shown in **Figure 1A**. Among them, *B7-1*, *B7-DC*, and *B7-H3* were up-regulated in LSCC tissues (absolute logFC≥2.0, adjusted p<0.05). To validate results from TCGA, we used the GEO data for LSCC patients. The results also indicated significant upregulation of *B7-1*, *B7-DC*, and *B7-H3 in LSCC* samples compared to normal levels (**Figure 1B**). Kaplan-Meier analysis showed that none of them was significantly correlated with OS (*P*>0.05, **Figure 1C**). However, patients with high B7-H3 expression had shorter OS time than patients with low B7-H3 expression.

### B7-H3 Is Overexpressed in LSCC Tissues

**Figure 2:**
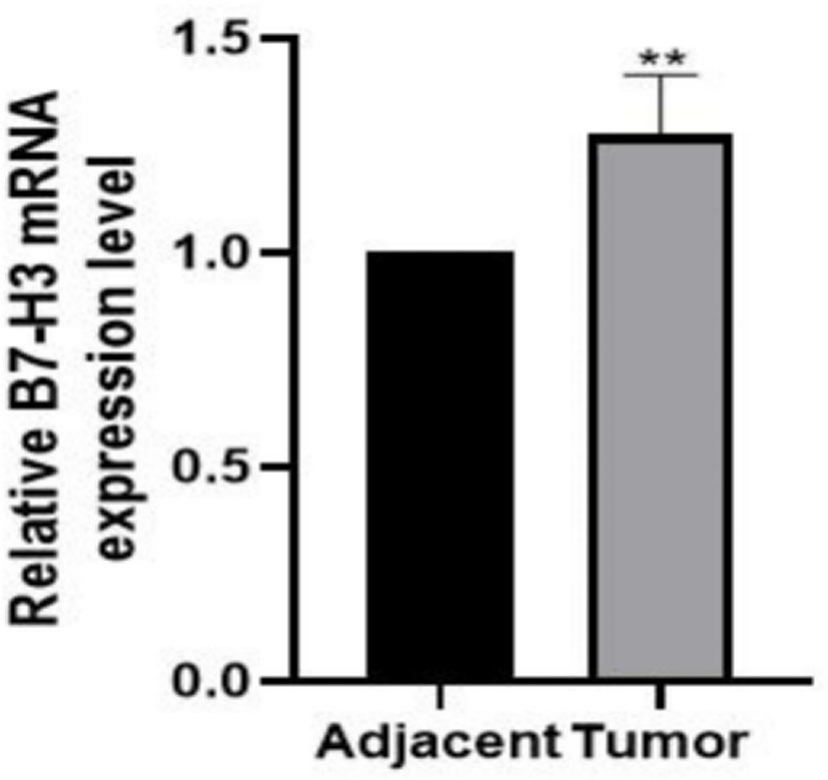
Overexpression of B7-H3 in LSCC tissues compared with noncancerous tissues. B7-H3 mRNA was tested by qRT-PCR, relative to the internal endogenous control GAPDH. B7-H3 show significant increase in expression in LSCC compared to noncancerous tissues. **p < 0.01 (n = 12).

Next, the mRNA expression level of B7-H3 in LSCC and adjacent normal tissues (n=12) was determined. Results showed that expression of B7-H3 mRNA was significantly higher in LSCC tissues than in adjacent normal tissues (*P*< 0.01, **Figure 2**).

**Figure 3:**
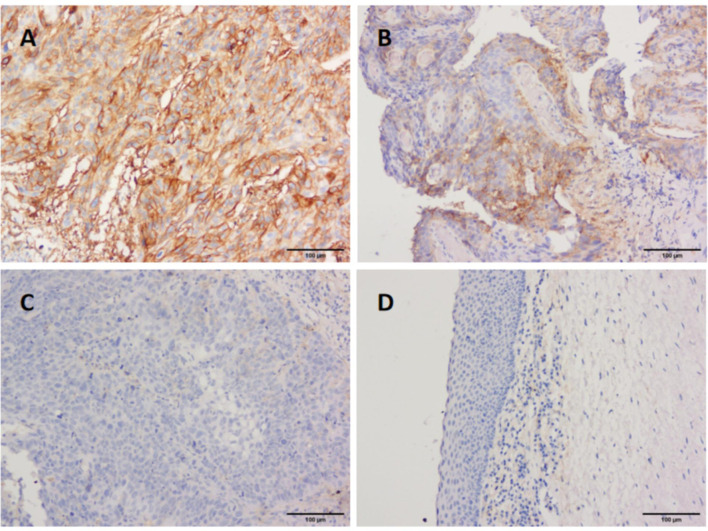
Immunohistological staining of LSCC tissues for B7-H3 expression. Representative tissues of, strong staining **(A)**, moderate **(B)**, weak **(C)**, and adjacent normal tissues **(D)** for B7-H3 expression in LSCC samples. (magnification ×200).

In addition, IHC was performed on 122 wax LSCC samples and 30 wax adjacent normal tissues to further evaluate the role of B7-H3. B7-H3 positive staining was mainly found in membranes of cancer cells and partially in the cytoplasm. However, central keratinized area of cancer nests was negatively stained. The positive expression of B7-H3 was also found in tumor-infiltrating immune cells. Results indicated that the expression of B7-H3 was significantly higher in LSCC tissues than in the adjacent normal tissues (**Figure 3**), suggesting that B7-H3 is overexpressed in LSCC.

### Correlation of B7-H3 Expression With Clinicopathological Features of LSCC Patients

Among the 122 LSCC cases, 75 (61.5%) stained strongly positive for B7-H3. **Table 1**, shows that the expression of B7-H3 in LSCC correlated with T stage, lymph node metastasis, and TNM stage (P<0.05), but not with age, sex, tumor location, pathological differentiation, alcohol consumption, smoking, or cancer recurrence.

**Table 1:** Clinicopathological factors associated with B7-H3 expression levels.

Clinicopathological Features	n	B7-H3 expression		P
		low	high	
		(≤3)	(>3)	
gender				
male	118	45	73	0.632
female	4	2	2
age				
<60	54	22	32	0.654
≥60	68	25	43
T stage				
T1+T2	86	38	48	0.047
T3+T4	36	9	27
lymph node metastasis				
N0	105	45	60	0.015
N+	17	2	15
TNM stage				
I+II	81	39	42	0.002
III+IV	41	8	33
pathological differentiation				
high	57	25	32	0.257
moderate,low	65	22	43
alcohol consumption				
positive	33	14	19	0.59
negative	89	33	56
smoking index				
<400	38	15	23	0.885
≥400	84	32	52
recurrence				
positive	17	6	11	0.768
negative	105	41	64

### Overexpression of B7-H3 Confers Poor Prognosis to LSCC Patients

**Figure 4:**
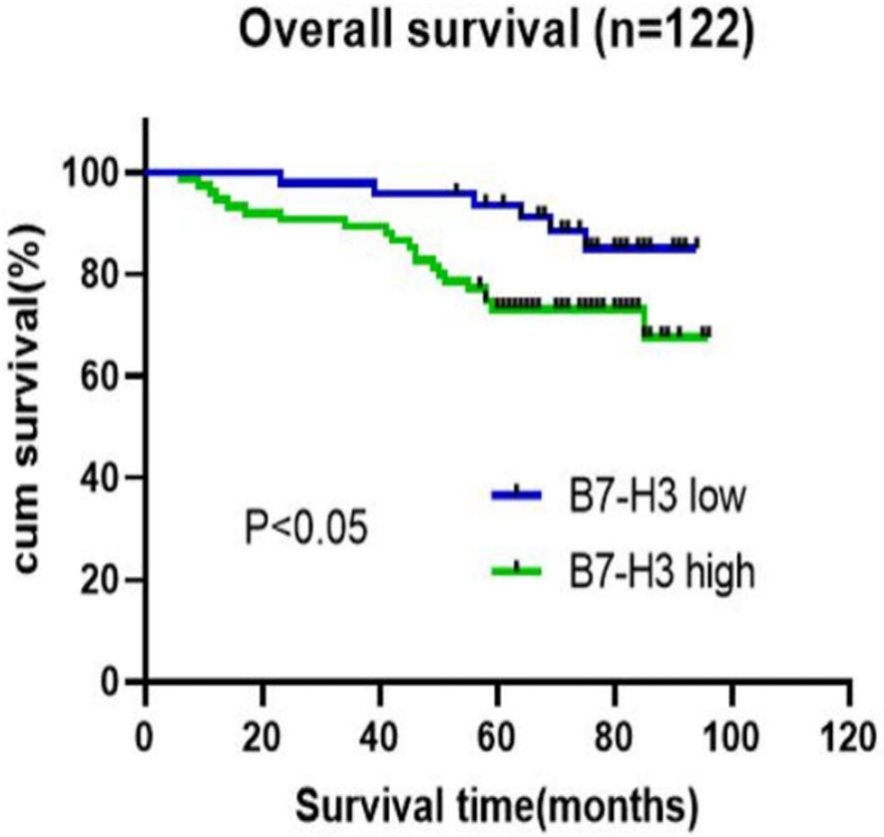
Survival analysis of correlation of overexpression of B7-H3 and overall survival in LSCC patients. The overall survival rate was calculated with Kaplan-Meier method and compared with log-rank test (n=122).

The median follow-up was 75.0 months in the total cohort. A Kaplan–Meier curve showed that overexpression of B7-H3 was significantly associated with poor prognosis at 60 months (*P* = 0.003, **Figure 4**). Next, univariate and multivariate Cox regression analyses were performed to determine whether B7-H3 is an independent prognostic factor for OS. Univariate analyses showed that T stage (P=0.039), lymph node metastasis (*P*=0.002), alcohol consumption (*P*=0.023), cancer recurrence (*P*=0.021), and B7-H3 expression (*P*=0.045) had a significant association with OS. On the other hand, multivariate analysis indicated that lymph node metastasis, alcohol consumption, cancer recurrence, and B7-H3 expression (*P*=0.038) were independent factors influencing the OS (**Table 2**). Collectively, these results suggest that overexpression of B7-H3 is significantly associated with poor prognosis.

**Table 2:** Univariate and Multivariate Cox proportional hazards analysis for overall survival in 122 LSCC patients.

Variable	Group	Univariate Cox regression		Multivariate Cox regression	
		HR (95%CI)	*P* value	HR (95%CI)	*P* value
age	>60 (*vs*.≤60)	0.789 (0.348-1.789)	0.259		
gender	Male (*vs*. female)	0.696 (0.094-5.172)	0.776		
smoking index	≥400 (*vs*.<400)	1.346 (0.53-3.413)	0.556		
pathological differentiation	well (*vs*.moderate,low)	1.735 (0.735-4.094)	0.303		
T stage	T3+T4 (*vs*. T1+T2)	2.182 (0.956-4.978)	0.039	1.774 (0.696-4.521)	0.23
lymph node metastasis	N+ (*vs*. N0)	3.809 (1.564-9.277)	0.002	5.708 (1.974-16.506)	0.001
alcohol consumption	Positive (*vs*. negative)	2.747 (1.204-6.267)	0.023	4.571 (1.829-11.425)	0.001
recurrence	Positive (*vs*. negative)	2.758 (1.134-6.71)	0.021	5.54 (2.036-15.079)	0.001
B7-H3	low (*vs*. strong)	3.423 (1.164-10.067)	0.045	3.08 (1.066-8.899)	0.038

HR, hazard ratio; CI, confidence interval.

### Relationship Between B7-H3 Expression and Infiltration of CD8^+^ T Cells

**Figure 5:**
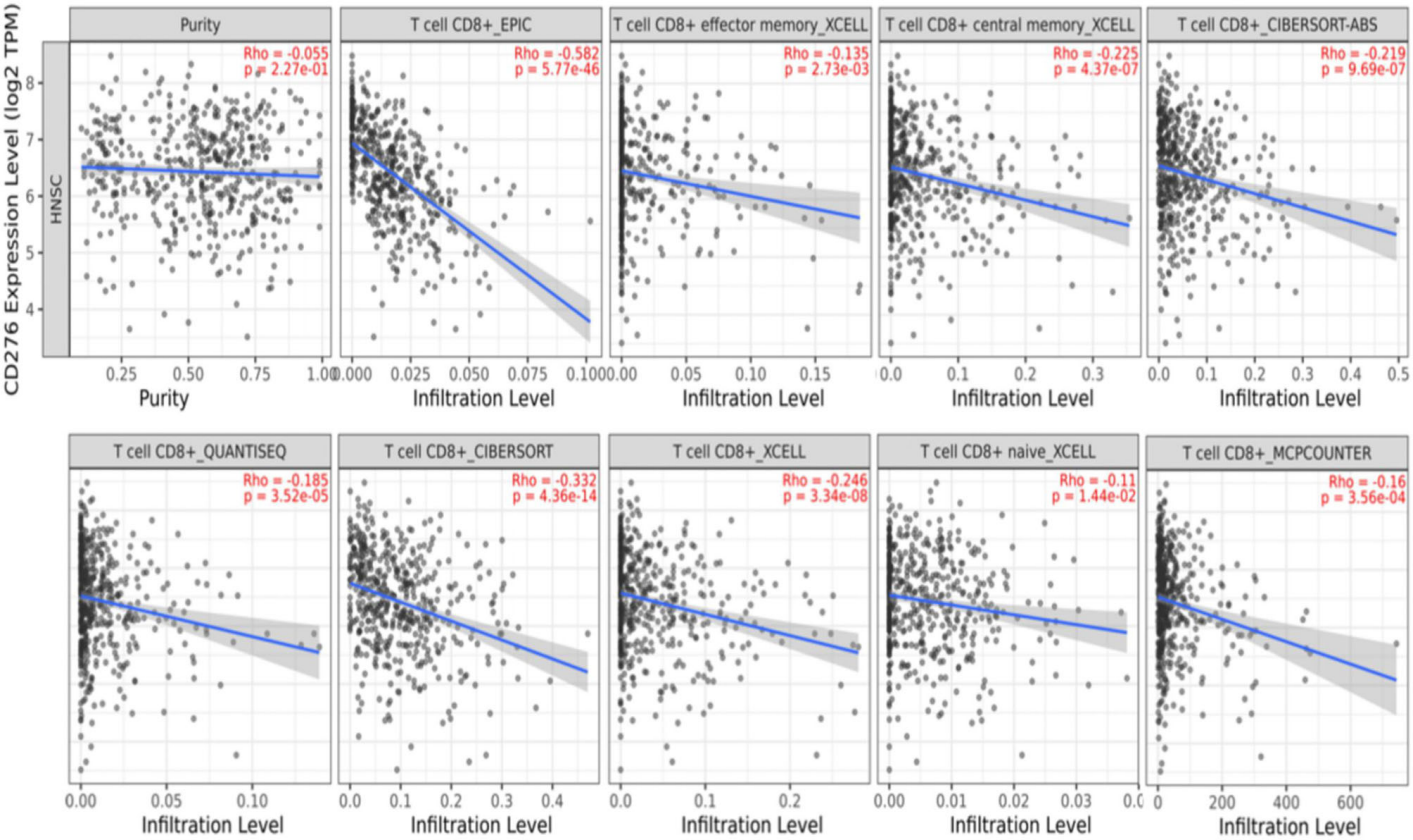
Correlation of B7-H3 expression with infiltration of CD8^+^ T cells. The data was obtained from TCGA HNSCC datasets and analyzed by 6 different algorithms from TIMER 2.0.

**Figure 6:**
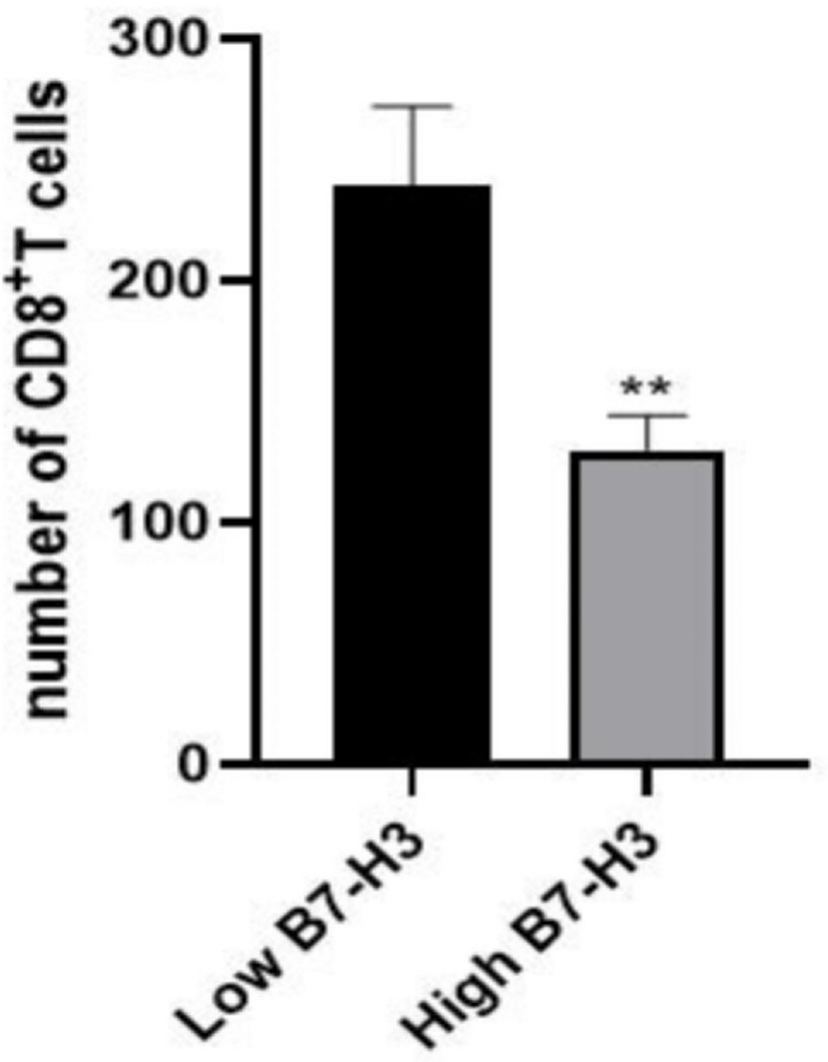
The number of tumor-infiltrating CD8^+^ T-cell was inversely correlated with B7-H3 expression in 24 patients with LSCC (**p < 0.01). This correlation was tested by Wilcoxon rank sum test.

**Figure 7:**
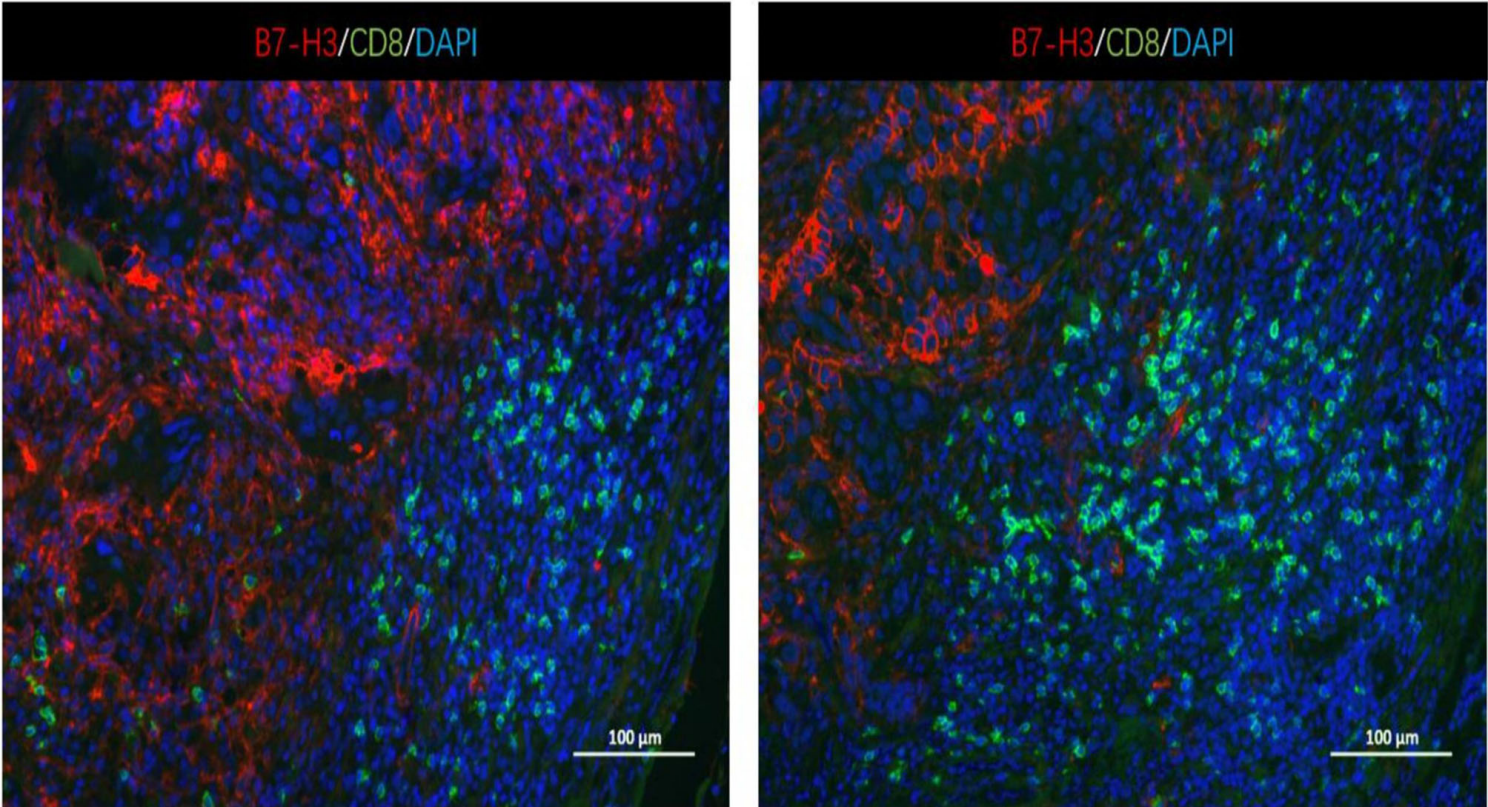
Representative immunofluorescence images of B7-H3 and tumor-infiltrating CD8^+^ T cells in LSCC tissues. Original magnification ×100; scale bar, 100 μm. No apparent colocalization of the B7-H3 (red) and CD8 (green) signal was found.

Furthermore, we analyzed a TCGA cohort with head and neck squamous cell carcinoma (HNSCC) individuals using six different algorithms and found that the expression of B7-H3 was inversely correlated with the infiltration level of CD8^+^ T cells (**Figure 5**). To determine the potential relationship between B7-H3 expression and tumor infiltrating CD8^+^ T cells in LSCC, immunohistochemical staining was performed in 24 cases for CD8. Results showed that the number of CD8^+^ T cells per 3HPF of high B7-H*3* expression cases ranged from 50 to 238 (mean ± SD: 130 ± 58.2), and the number of low B7-H3 expression cases ranged from 111 to 381 (mean ± SD: 239 ± 87.0), indicating significant differences (**Figure 6**). Given that previous IHC results showed a positive expression of B7-H3 in tumor-infiltrating immune cells, immunofluorescence was further performed to examine B7-H3 expression in CD8^+^ T cells. The results revealed a trend toward higher stroma CD8^+^ T-cell infiltration in the tumors relative to lower B7-H3 expression (**Figure 7**). However, B7-H3 was not detected in CD8^+^ T cells. Collectively, these results suggest that B7-H3 is negatively correlated with CD8^+^ T-cell infiltrate density.

## Discussion

Previous studies have reported that B7 family ligands are overexpressed in many types of malignancies and B7-H3 is highly expressed in HNSCC (12–14). This study examined the expression of a set of B7 family genes in LSCC tissues using TCGA and GEO cohorts. It was found that three DEGs including B7-H3 were upregulated in LSCC tissues. According to our PCR analysis, B7-H3 mRNA was also overexpressed in LSCC tissues. Furthermore, results of IHC test revealed that high B7-H3 expression level was associated with advanced T stage, lymph node metastasis, and advanced TNM stage. Moreover, high B7-H3 expression level was correlated with poor prognosis of LSCC. To the best of our knowledge, this study provides the first evidence that overexpression of B7-H3 can be used to predict the prognosis of patients with LSCC.

As a novel member of B7 family ligands, B7-H3 has been identified as an immune checkpoint in many types of cancers. It has been detected in several cancers, including melanoma, oral squamous carcinoma, colorectal cancer, and numerous cancers (10, 15–22). Overexpression of B7-H3 is associated with poor prognosis in most cancers. Previous studies demonstrated that B7-H3 was highly expressed in HNSCC tissues and thus it may be a prognostic marker (5, 14, 16). However, the role of B7-H3 in LSCC has not been elucidated. In this study, we investigated the expression of B7-H3 in LSCC tissues, and found that B7-H3 was extensively expressed on membranes of cancer cells and slightly expressed in the cytoplasm. In addition, B7-H3 was highly expressed at the invasive front of tumor. Recent studies have reported that high expression of B7-H3 is associated with adverse clinicopathologic features and poor survival. In the present study, B7-H3 was found to be associated with advanced T stage, lymph node metastasis, and advanced TNM stage (III-IV), indicating that B7-H3 may regulate LSCC progression. However, B7-H3 was not correlated with recurrence in LSCC, unlike the results obtained for glioblastoma (23) and some other cancers (24, 25). Therefore, we will continue to follow up these patients to observe the possibility of recurrence. Next, the ability of B7-H3 to predict the prognosis of patients was tested. Results showed that B7-H3 was significantly associated with poor prognosis, suggesting that it might be an independent predictor of OS. Overall, this study reveals that B7-H3 may be used as a marker for predicting the prognosis of LSCC. While, as the adjacent tumor sample cohort size was limited, larger cohorts are required to validate these observations in the future.

As an immune checkpoint, B7-H3 acts as a costimulatory and coinhibitory molecule that influence T cell proliferation (26). Chapoval et al. (3) reported that B7-H3 promoted proliferation of both CD4^+^ and CD8^+^ T-cells, and enhanced function of the cytotoxic T-cell (CTL). However, some studies have shown that B7-H3 has a negative effect on proliferation of T-cells (27, 28). Similarly, B7-H3 was inversely associated with the infiltration of CD8^+^ T cells, and B7-H3 blockade decreased the number of cancer stem cells in HNSCC (29). In this study, IHC staining results revealed that immune cells in the stromal tissue stained positive for B7-H3. To further determine the relationship between B7-H3 and CD8^+^ T cell infiltration, immuno-fluorescence staining experiment was done and tissues were examined under a confocal microscope. It was found that CD8^+^ infiltration was mainly occurred in stromal areas and positive staining for B7-H3 was mostly seen in tumor tissues. In addition, low B7-H3 expression was associated with high CD8^+^ infiltration. However, B7-H3 was not expressed on CD8^+^ T cells. Similar results were obtained from IHC staining tests. CD8^+^ T lymphocytes have been shown to be correlated with good prognosis in many cancer types and may be an independent favorable prognostic factor in HNSCC (30). Based on results of this study and previous studies (4, 19, 25), we infer that B7-H3 might inhibit proliferation of CD8^+^ T cells and impair antitumor immunity in LSCC. However, the specific mechanism of B7-H3 regulating CD8^+^ T cells infiltration needs further study.

In this study, although B7-H3 was found to be highly expressed in LSCC and associated with poor prognosis, the underlying molecular mechanism was not elucidated. However, we hypothesized that B7-H3 may inhibit CD8^+^T cells infiltration, hereby promote tumor immune escape. Similarly, a recent study reported that inhibition of B7-H3×m4-1BB suppressed tumor growth and increased the number of terminally differentiated CD8^+^ T cells in tumor models (31). B7-H3 was also found to be an inhibitor of CD4^+^T-cells, Th cells, Treg cells, and NK cells (32, 33). However, the receptor of B7-H3 is currently unknown. Some researchers have proposed that the triggering receptor expressed on myeloid cell (TREM)-like transcript 2 (TLT-2) may be a putative counter receptor for B7-H3 (34, 35). Therefore, further studies are advocated to identify the receptor of B7-H3, and elucidate the signaling mechanism of B7-H3. Immunotherapy has emerged as a promising treatment for malignancies. PD-L1/PD-1 blockade therapy has been approved for advanced head and neck cancer treatment, but a large fraction of patients do not respond to this therapy. Other studies have investigated the use of B7-H3 as an inhibitor of antibodies. For instance, 8H9, ^131^I-monoclonal antibodies targeting B7-H3, was found to be an immunotherapeutic agent in neuroectodermal tumors, and has been tested in a phase I trial to treat patients with diffuse intrinsic pontine glioma (36, 37). Therefore, this study suggests that B7-H3 may be a promising target for LSCC immunotherapy.

## Conclusions

Overall, this study demonstrates that B7-H3 is upregulated in LSCC, and the high expression level of B7-H3 was closely associated with clinicopathological features and poor prognosis of LSCC patients. Moreover, B7-H3 may inhibit CD8^+^T-cell infiltration. These results suggest that B7-H3 may be a critical biomarker for predicting LSCC prognosis and a new target for LSCC immunotherapy.

## Data Availability Statement

The datasets presented in this study can be found in online repositories, further inquiries can be directed to the corresponding authors.

## Ethics Statement

The studies involving human participants were reviewed and approved by Ethical Committee of Sun Yat-Sen University. The patients/participants provided their written informed consent to participate in this study.

## Author Contributions

YL performed the experiments and contributed to the writing of the manuscript. QC was as the cooperation teacher of the experiments and took charge of supplementary bioinformatic analysis; XS performed the experiments. XC took charge of the result analysis; ZG played a guiding role in conceiving and designing the study,and provided the overall writing guidance. All authors reviewed the manuscript. All authors contributed to the article and approved the submitted version.

## Funding

This study was supported by the National Natural Science Foundation of China No. 81572648.

## Conflict of Interest

The authors declare that the research was conducted in the absence of any commercial or financial relationships that could be construed as a potential conflict of interest.

## Publisher’s Note

All claims expressed in this article are solely those of the authors and do not necessarily represent those of their affiliated organizations, or those of the publisher, the editors and the reviewers. Any product that may be evaluated in this article, or claim that may be made by its manufacturer, is not guaranteed or endorsed by the publisher.
